# Editorial: Gut microbe and colorectal cancer

**DOI:** 10.3389/fcimb.2023.1247619

**Published:** 2023-07-04

**Authors:** Yugen Chen

**Affiliations:** Department of Colorectal Surgery, Affiliated Hospital of Nanjing University of Chinese Medicine, Nanjing, China

**Keywords:** colorectal cancer, gut microbiota, epidemiological investigation, diagnosis, pathological mechanism, therapeutic interventions, editorial

Colorectal cancer (CRC) is a significant global health concern, due to its increasing incidence worldwide. Recent research has proven that the gut microbiota plays a crucial role in the development and progression of CRC. Numerous studies have explored the relationship between gut microbiota and CRC, shedding light on potential diagnostic, mechanistic, and therapeutic applications. This special Research Topic aims to gather recent insights into influences of gut microbiota on CRC, which includes epidemiological investigation, diagnosis, pathological mechanism and therapeutic interventions.

The first article by Xiong et al. focused on the differences in gut microbiota profiles between patients with early-onset colorectal cancer (EOCRC) and late-onset colorectal cancer (LOCRC). The results demonstrated that patients with EOCRC exhibited a distinct gut microbiota composition compared to those with LOCRC. This dysbiosis was reflected in the species diversity, abundance of bacteria, and abnormal functional predictions. This study adds to the growing body of work on role of gut microbiota as a potential diagnostic tool for distinguishing between different types of CRC.

Chen et al. aimed to identify the gut microbiota associated with lymph-vascular invasion (LVI) in CRC patients and construct predictive labels for accurate assessment of LVI. The results revealed significant differences in the composition, abundance, and biological function of the intestinal microbiome between patients with and without LVI. The study also successfully constructed predictive models based on microbial markers, which could aid in predicting LVI in CRC patients. These findings have important implications for improving the diagnosis and prognosis of CRC.

The contribution by Yang et al. examined the global research output and trends in the crosstalk between gut microbiota and CRC. The analysis revealed a rapid increase in publications on CRC research involving in gut microbiota, with China and the United States leading in terms of scientific contributions. The study identified hotspots in the field, including the effect of gut microbiota on tumorigenesis, CRC treatment, screening, and the underlying mechanisms. These findings highlight the importance of ongoing research in understanding the relationship between gut microbiota and CRC.

Zheng et al. focused on the implication of gut microbiota in recovery from gastrointestinal surgery. The disruption of gut microbiota during the perioperative period can contribute to postoperative complications. Understanding how to balance gut microbiota is crucial for enhancing recovery from gastrointestinal surgery. This study emphasized the importance of preserving the beneficial functions of gut microbiota in the recovery from gastrointestinal surgery.

In the paper of this Research Topic, Gu et al. reviewed the role of gut microbiota and bacterial metabolites in mucins of CRC. The crosstalk between host mucins and gut microbiota was found to be associated with the occurrence and development of CRC. Dysbiosis of gut microbiota can lead to dysfunction of mucins, potentially triggering host responses and the progression of CRC. These conclusions further highlight novel treatment strategies targeting gut microbiota and bacterial metabolites as new avenues for CRC therapy.

The final research by Fan et al. explored the potential of Traditional Chinese medicine (TCM) in targeting gut microbiota for the treatment of CRC. TCM was highlighted as a promising source of natural medicines and herbal products with anti-CRC properties. The application of TCM to modulate gut microbiota represents an exciting area of investigation for CRC management. This article emphasizes the potential of TCM as a promising approach for targeting gut microbes in the treatment of CRC, inspiring future research in this field.

In conclusion, this diverse Research Topic of primary research papers provide valuable insights into the role of gut microbiota in CRC. The long-term goal of these research efforts are to further elucidate the crucial role of potential probiotics and pathogenic bacteria in the development and progression of CRC. Additionally, the investigation of specific protein components or metabolites derived from microbiota, as well as the interaction between gut microbiota and TCM, holds great potential as a promising direction for in-depth research on the intricate relationship between gut microbiota and CRC. Exploring these factors can provide valuable insights into the mechanisms and diagnosis underlying CRC pathogenesis and potentially contribute to the development of novel therapeutic strategies targeting the gut microbiota.

## Author contributions

The author confirms being the sole contributor of this work and has approved it for publication.

